# A qualitative approach to examining antimicrobial prescribing in the outpatient dental setting

**DOI:** 10.1017/ash.2022.242

**Published:** 2022-06-24

**Authors:** Ashley M. Hughes, Charlesnika T. Evans, Margaret A. Fitzpatrick, Ibuola O. Kale, Amanda Vivo, Taylor L. Boyer, Pooja A. Solanki, Gretchen Gibson, M. Marianne Jurasic, Lisa K. Sharp, Kelly L. Echevarria, Katie J. Suda

**Affiliations:** 1 Department of Biomedical and Health Information Sciences, University of Illinois Chicago, Chicago, Illinois; 2 Center of Innovation for Complex Chronic Healthcare, Edward Hines, Jr. VA Hospital, US Department of VA, Hines, Illinois; 3 Department of Preventive Medicine and Center for Health Services & Outcomes Research, Institute for Public Health and Medicine, Feinberg School of Medicine, Northwestern University, Chicago, Illinois; 4 Division of Infectious Diseases, Department of Medicine, Stritch School of Medicine, Loyola University Chicago, Maywood, Illinois; 5 Center for Health Equity Research and Promotion, VA Pittsburgh Healthcare System, US Department of Veterans’ Affairs, Pittsburgh, Pennsylvania; 6 Oral Health Quality Group, Veterans Health Administration, Office of Dentistry, Washington, D.C.; 7 Boston University Henry M. Goldman School of Dental Medicine, Boston, Massachusetts; 8 VA Center for Healthcare Organization & Implementation Research, Edith Nourse Rogers Memorial Veterans’ Hospital, Bedford, Massachusetts; 9 Department of Pharmacy Systems Outcomes and Policy, University of Illinois Chicago, Chicago, Illinois; 10 VA Pharmacy Benefits Management Services, San Antonio, Texas; 11 College of Medicine, University of Pittsburgh, Pittsburgh, Pennsylvania

## Abstract

**Objective:**

To understand barriers and facilitators to evidence-based prescribing of antibiotics in the outpatient dental setting.

**Design:**

Semistructured interviews.

**Setting:**

Outpatient dental setting.

**Participants:**

Dentists from 40 Veterans’ Health Administration (VA) facilities across the United States.

**Methods:**

Dentists were identified based on their prescribing patterns and were recruited to participate in a semistructured interview on perceptions toward prescribing. All interviews were recorded, transcribed, and double-coded for analysis, with high reliability between coders. We identified general trends using the theoretical domains framework and mapped overarching themes onto the behavior change wheel to identify prospective interventions that improve evidence-based prescribing.

**Results:**

In total, 90 dentists participated in our study. The following barriers and facilitators to evidence-based prescribing emerged as impacts on a dentist’s decision making on prescribing an antibiotic: access to resources, social influence of peers and other care providers, clinical judgment, beliefs about consequences, local features of the clinic setting, and beliefs about capabilities.

**Conclusions:**

Findings from this work reveal the need to increase awareness of up-to-date antibiotic prescribing behaviors in dentistry and may inform the best antimicrobial stewardship interventions to support dentists’ ongoing professional development and improve evidence-based prescribing.

Antibiotic resistance is a global health crisis, with human healthcare consumption leading as an undeniable factor in the rise of antimicrobial resistance (AMR).^1^ Multiple guidelines have been developed to improve evidence-based antibiotic prescribing.^1,2^ Among millions of antibiotic prescriptions each year, most take place in the outpatient setting. Most outpatient antibiotic prescriptions are not evidence based or clinically indicated,^3,4^ which contributes to AMR.

Dentists prescribe 6%–12% of all antibiotics in the outpatient setting,^3^ accounting for millions of antibiotic courses each year.^5–8^ Furthermore, dentists rank fourth in antibiotic prescribing in the United States per capita,^9^ making them a significant source of outpatient prescribing. Nevertheless, the contribution of dentists on overall antibiotic use has only recently been recognized. Current data show areas in which antibiotic prescribing is suboptimal, highlighting a need for dental antimicrobial stewardship interventions. Given the lack of knowledge about why and what dentists prescribe, we sought to identify barriers and facilitators to evidence-based prescribing and identify targeted behavioral interventions to improve antibiotic use. Coupled with a high proportion of guideline-discordant prescribing,^10^ there is a burgeoning need to uncover and address the root causes of inappropriate antibiotic prescribing, which may be specific to the outpatient dental care setting.

In this study, we examined dental prescribing of antibiotics through the lens of behavior change, focusing on barriers and facilitators to evidence-based prescribing.

## Methods

### Study design and setting

This study had a cross-sectional, qualitative design. We used interviews with dentists practicing in the Veterans’ Health Administration (VHA) to gain insight into the factors that facilitate or impede evidence-based antibiotic prescribing.

### Interview guide development

The semistructured interview guide underwent several iterations prior to data collection. The initial set of questions were developed through multidisciplinary discussions with physicians, pharmacists, public health experts, and VHA dentists. Questions were then revised to elicit specific components of the theoretical domains framework^11,12^ and were pilot tested with 2 VHA dentists. The resulting interview guide was then tested with a third VHA dentist via a mock interview for comprehension and face validity (see Appendix A). Materials were reviewed and approved by all members of the study team, and the study received approval from the Hines VA Institutional Review Board (no. 17-051).

### Site selection

Recruitment sites were identified using prescribing trends across VHA dental clinics via the Corporate Data Warehouse (CDW), a national repository of medical and dental information from 170 VHA medical centers (VAMCs) and 1,074 outpatient sites. Sites were categorized into lower 75th and upper 25th percentiles based on their visit-based antibiotic prescription rates (no. of prescriptions per 100 visits) by dentists. The 5 highest prescribing facilities (defined by the upper 25^th^ percentile) and 5 lowest prescribing facilities (lower 75^th^ percentile) were purposely sampled in each of the 4 US Census Bureau regions for interview recruitment. Overall, 40 sites were included in the sampling frame.

### Recruitment

Chiefs of dentistry at selected sites received an e-mail informing them of the project and the support of the VHA Office of Dentistry. The e-mail did not include antibiotic prescribing rates.

From 40 sites, dentists who prescribed at least one antibiotic (2016–2017) were invited to participate in the study. Trained research assistants contacted eligible dentists by first sending an e-mail with an informational opt-out letter. Then, dentists were contacted using e-mail, phone, and then instant messages and phone calls. A maximum of 3 contact attempts were allowed per recruitment approach (eg, maximum of 3 phone calls and 3 Skype messages).

**Fig. 1.**
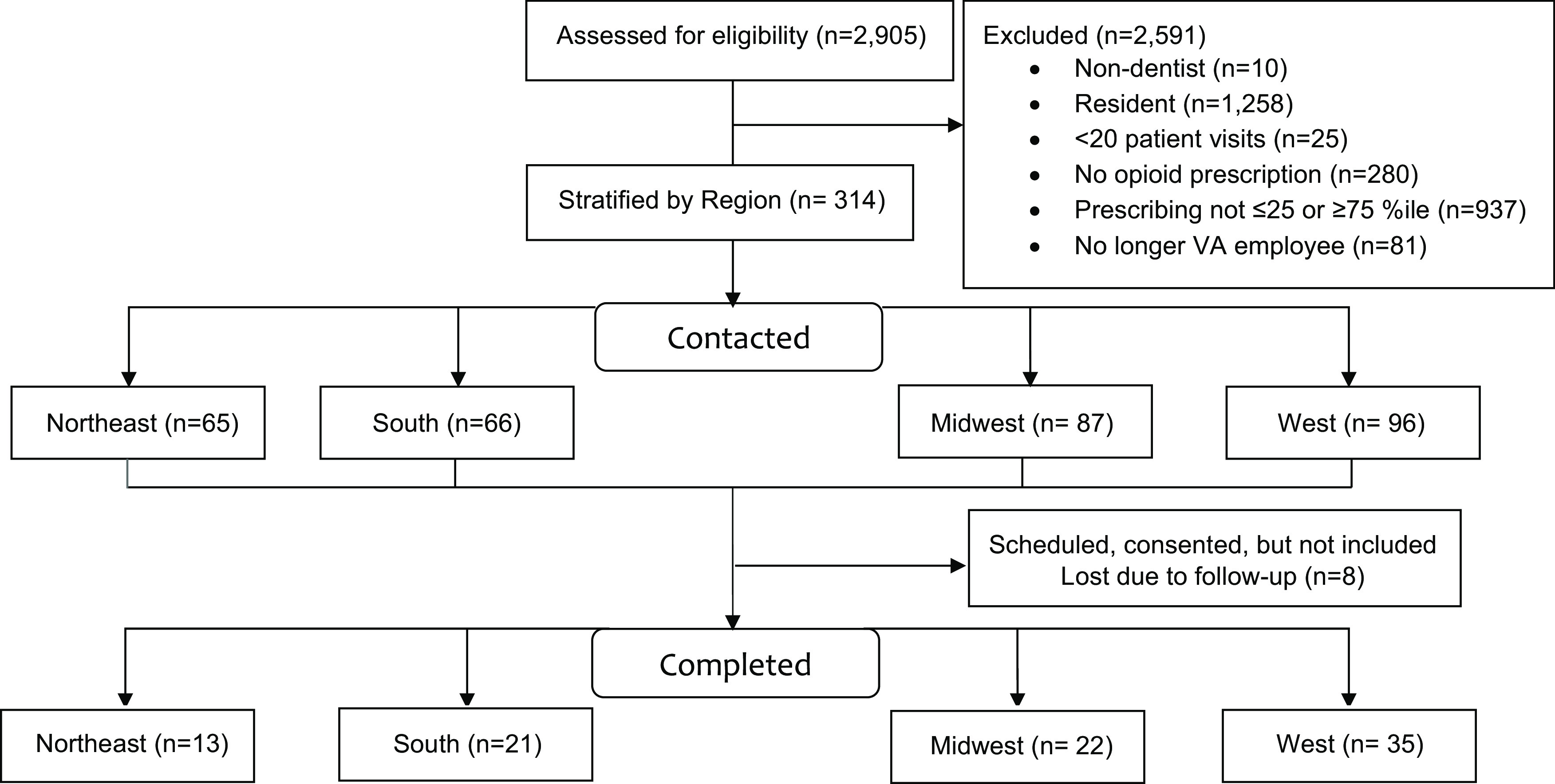
Recruitment flow diagram.

Dentists who agreed to participate scheduled a day and time for the interview and joined the interview via an audio-only call. In the few cases in which dentists were lost to follow-up (eg, rescheduling interview, not identifying a day or time), the study team made 2 follow-up attempts to schedule or reschedule an interview (Fig. 1). Up to 7 dentists per facility could participate in an interview; high and low prescribing rates reported at the prescriber level were used to prioritize participant recruitment at each site (ie, high or low prescribers nationally) (Appendix B).

### Data collection

Study procedures were explained to participants, and informed consent was obtained verbally and was recorded. Audio-recording of consent was mandatory for study participation, but recording of the interviews was optional and was performed only with participant consent. In the absence of an audio-recorded interview, research assistants took field notes (n = 2). The first 5 interviews involved at least 1 member of the investigative personnel with expertise in qualitative approaches (A.M.H. and L.K.S.), and they were reviewed for accuracy by content experts (K.J.S. and C.T.E.). A qualitative expert (A.M.H. and L.K.S.) met weekly with interviewers. Per VHA guidelines, participating dentists received no reimbursement. All audio-recorded interviews were transcribed verbatim, and all identifying information was removed. Self-reported demographics for dentists were stored separately from transcripts and field notes.

### Analysis

De-identified transcripts and field notes were uploaded to NVivo version 12 software (QSR International, Doncaster, Australia) for qualitative analysis.^13^ We used thematic content analysis to extract themes and trends informed by the theoretical domains framework, further organizing themes into three overarching categories using a related framework, the “Capabilities, Opportunities, and Motivations”-Behavior (COM-B) model. COM-B categories and associated themes informed by the theoretical domains framework have been shown to influence prescribing behavior within and outside dentistry.^11^

### Coding and interrater reliability

A codebook was developed iteratively by first reviewing a subset 5 transcripts individually (P.S., T.L.B., L.K.S., and A.M.H.), followed by discussion of potential codes and rules. Once codebook rules were devised and tested, the remaining transcripts were coded independently by at least 2 trained members of the research team (A.V. and I.O.K.), who met regularly to identify and resolve discrepancies. Coders were in high agreement (99.9%; *K* = .97) and resolved any discrepancies via consensus for 100% agreement. A third coder (A.M.H.) was present in the event that consensus could not be reached. Upon completion of coding, codes were reviewed for consistency and sensemaking by 3 members of the study team (A.V., I.O.K., and A.M.H.). Themes were extracted and identified to describe results as they pertain to overall evidence-based antibiotic prescribing practices.

## Results

In total, 98 dentists agreed to participate, but 8 were lost due to follow-up and/or difficulty in scheduling an interview, for a final sample of 90 dentists who were interviewed. Among them, 58 respondents (65.9%) were white, 52 (57.8%) were male, and 72 (80.0%) were general dentists (ie, nonspecialty dentists). Participants had an average of 20 years of experience practicing dentistry, excluding residency (SD, 11.8; median, 19 years; interquartile range [IQR], 9–30). Although most dentists practiced within a predominantly urban area (n = 56, 62.2%), the sample proportionately represented the Northeast (n = 13, 14.4%), South (n = 21, 23.3%), Midwest (n = 22, 24.4%), and Western census regions (n = 34, 37.9%,) of the United States (Table 1).

**Table 1.** Sample Demographics (n=90)

Characteristic	No. (%)
**Sex**	
Male	52 (57.78)
Female	38 (42.22)
**Race/Ethnicity^a^**	
White	58 (65.91)
Asian	19 (21.59)
Black	5 (5.68)
Hispanic	6 (6.82)
Prefer not to share	2
**Dentist type**	
General	72 (80)
Specialty	18 (20)
**Years practicing in VAMC** Mean (SD) Median (Q1–Q3)	20 (11.76) 19 (9–30)
**Region**	
West	34 (37.78)
Midwest	22 (24.44)
South	21 (23.33)
Northeast	13 (14.44)
**Location of VAMC**	
Urban	56 (62.22)
Suburban	25 (27.78)
Rural	9 (10)

Note. SD, standard deviation; VAMC, Veterans’ Affairs medical center.

a As dentists reported ‘all that apply’ for race/ethnicity, percentages may not equal 100% when summed. Additionally, 2 participants declined to answer this question.

### Capabilities

**Fig. 2.**
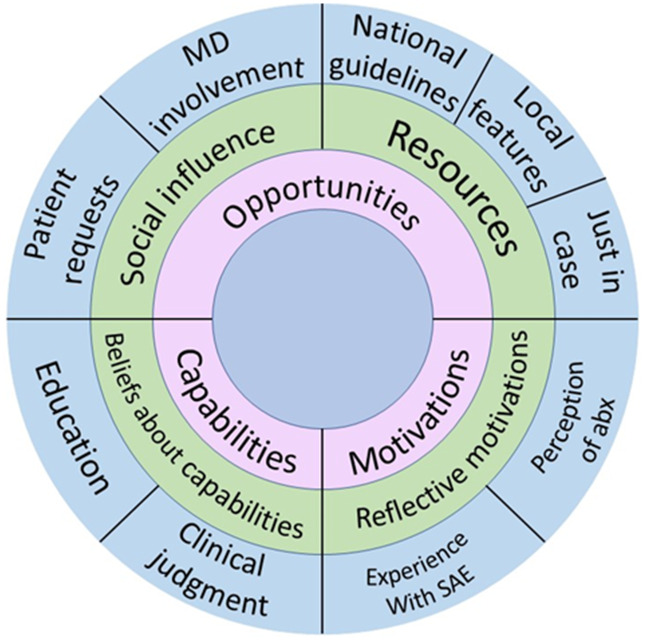
Behavior change wheel.

Capabilities encompass an individual’s capacity, both physical and psychological, to engage in the activity of antibiotic prescribing (Table 2).^14^ Capabilities typically include both physical (eg, physical strength) and psychological components (eg, knowledge). However, our analysis revealed that primarily cognitive capabilities were relevant to appropriate dental prescribing of antibiotics (Fig. 2).

**Table 2.** COM-B and TDF Components With Example Quotes

Com-B Component	Domain(s) of the TDF	Themes	Frequency, No.	Sample Quote
Capabilities	Beliefs about capabilities	Sufficient knowledge	154	“I don’t think you ever have sufficient knowledge. You can always learn.” -GD-01-37
		Clinical judgment	168	“The way they present clinically, first and foremost. Secondly, their medical histories. Thirdly their propensity for infection based upon their general medical condition.” –GD-03-72
Opportunities	Social influence	Physician influence	119	“Usually, *we defer to the medical provider*. According to the way it is stated, when a patient needs a premedication for a dental procedure because of a medical condition, it’s really the responsibility of the physician to order then. Now, most of the time it falls back on the dentist to order antibiotics.” –GD-02-47
		Patient requests	159	“They think they need them for everything! At least once or twice a week… with a lot of humor.” “It’s happened where patients aren’t educated of what’s going on in their mouth and they have asked but it’s rare-really rare. I would say once a year would a patient request an antibiotic rather than me simply explaining that they’ll need it.” –GD-01-04
	Resources	National guidelines	165	“Ay yi yi. I don’t know the year. I’m really bad with these, but I know that when-when I was in dental school we used to give pre-med before and after and that changed and now we just for example premedication for patients that have conditions that would effect would be affected by bacteria in the blood caused by dental procedures they changed it to only 2 grams before dental treatment 1 hour before. So I know like for example specifically for antibiotic pre-med has changed and then the preop the prescription of medication for infection. I don’t know that those have changed but I think those have been pretty steady and I just follow the recommended guidelines for each medication.” –SP-04-42
		Local resources	118	“Yeah, the more information I have [from the integrated EHR], I feel more confident in what I’m prescribing. Absolutely.” –GD-02-95
Motivations	Reflective motivations	Awareness	159	“Okay, dentistry wise, I feel like they do a really good job following the most current recommended guidelines from both the ADA and board of orthopedic surgeons. In general, I would say probably more moderate. I do know our population here at the VA is generally on the sicker side compared to private practice. I think they generally or moderately go within guidelines.” “– SP-04-44
		Experience with severe adverse events	160	“It doesn’t really change anything because I’ve never had anyone go into anaphylactic shock reaction. We can all have allergies. Stomach upset? Ok. A little rash? Ok. We document it and put it in their chart.” –GD-03-49

### Cognitive capabilities

Dentists expressed cognitive capabilities that enabled them to prescribe appropriately. These self-reported cognitive capabilities fell broadly into either knowledge of antibiotics or clinical judgement as a decision-making process. Clinical judgement involved details as to when, how much, and what to prescribe for antibiotics. Overall, participants perceived themselves to be knowledgeable and to prescribe according to evidence-based practices. Most dentists expressed confidence in having sufficient knowledge to handle patient requests for antibiotics.

Among all participating dentists, 82 (91.2%) noted the importance of patient-related factors, both clinical and nonclinical, in exercising their clinical judgement. Dentists noted numerous clinical factors, such as patient susceptibility to infection due to comorbid diseases and patient history (eg, allergies) as influencing their judgement (Table 3, Capabilities, no. 1).

**Table 3.** Quotes Referenced in the Results Section

No.	COM-B	Theme, Subtheme	Quote
1	Capabilities	Cognitive capabilities, clinical judgement	“…The transplant [patients] who have organ transplant. They have an increased risk of infection, immunosuppressed for any other reason other than diabetes, patients with cancer going through chemotherapy, maybe any other clinical conditions that can warrant that the patient may be at a high risk of infection from a dental procedure.” –GD-01-51.
2			“…We do a lot of surgery, so, the treatment we provide dictates sometimes if we give antibiotics. When I was in dental school, we did not give antibiotics routinely following certain types of extractions. Now, our oral surgeon recommends routine antibiotics for anytime you lay a flap. So, if you’re doing a surgical extraction where you’re laying a flap or implant placement when you’re laying a flap, they recommend doing a short course of antibiotics afterwards. So, we do that based on the treatment.” –GD-01-25
3	Opportunities	Resources, national guidelines	“I think we, yeah, we-we try and use all, you know, all VA guidelines as they apply to dentistry and as they apply to having patients that also are patients in other areas of the hospital. So, the prescribing guidelines are pretty much those guidelines that are recommended by the VA in addition to any American Dental Association recommended guidelines and the adjustments made are adjustments made according to if the patient is being seen by other physicians in the hospital and what medications they are taking.” –GD-02-31
4			“Yes, most of the questions come in when we use antibiotics for prophylaxis. Let’s say patients who have joint replacements, heart valve replaced, or transplant- that we use the national guidelines.” –GD-01-51
5		Resources, local resources	“…What’s really nice is if we order something, the pharmacy has set up a lot of alerts for us. So, if we do recommend something and potentially there’s either a potential for an allergic reaction or a cross reaction with other medications, we get alerted right away so that’s nice to have.” –GD-02-47
6		Social influence	“… there is a number of orthopedic surgeons in this region that give antibiotics for life that have routine joint replacement and, in those cases, if they don’t fit the criteria, I explain it to the patient and tell them we don’t provide that anymore and if they want it, they can get it from their physician. – GD-03-58
7		Social influence, physician influence	“I’ll write the antibiotic as recommended by the physician because I’m going to assume that they know what they’re doing. I don’t know if it’s right or wrong but I’m happy to do that.” -GD-03-58
8			“… I’ll be honest, I typically go with the recommendation. We have in the past forwarded updated guidelines to medical practitioners and sometimes it’s not always well received.” -GD-02-95
9		Social influence, physician influence pushback (low prescriber)	“The one I push back on is there is a number of orthopedic surgeons in this region that give antibiotics for life that have routine joint replacement and in those cases, if they don’t fit the criteria, I explain it to the patient…” - GD-03-58
10		Social influence, physician influence deferral (high prescriber)	“I defer to the physician if it’s related to a condition that the physician is treating…” – GD-04-87
11		Social influence, requests from patients	“Usually, when it’s not required in their case, they agree.” – GD-03-58
12	Motivations	Awareness and severe adverse events awareness	“Deliberately reduced our antibiotic prescriptions as it relates to orthopedic joint replacements, knees, and hips and I know our site in particular I think reduced our antibiotic premed prescription by almost 90% last year.” -GD-02-41

Invasiveness of the dental procedures were also considered as part of clinical judgment in cases of antibiotic prophylaxis. Dentists noted that invasiveness of dental procedures matters in prescribing a prophylactic antibiotic (Table 3, Capabilities, no. 2).

Interestingly, nonclinical factors, such as the patient accessibility to healthcare services due to location, day of the week, and/or weather, also influenced the dentist’s decision to prescribe an antibiotic. Part of the dentist’s clinical judgement is whether they engaged in ‘just in case’ prescribing (ie, prescribing antibiotics on an ‘as needed’ basis, with the patient deciding whether to take the antibiotics). Moreover, 82 dentists (91.2%) said that they did prescribe on a ‘just in case’ basis, meaning that most dentists engaged in this practice. Among those who engaged in ‘just in case’ prescribing practice, the behavior was justified with rationale of both clinical and nonclinical factors. For instance, several dentists attributed this decision to ambiguous clinical indication in the presence of concern related to patient circumstances, such as healthcare access (eg, patient physical limitations, elderly).

### Opportunities

Opportunities refer to the physical and social environment that contribute to antibiotic prescribing.^11,15^ Interestingly, this category included several sources of influence on dentist antibiotic prescribing behavior.

### Resources

Dentists reported using several resources to inform their antibiotic prescribing decisions. Among the available resources, national guidelines were noted as being helpful overall in adhering to evidence-based prescribing. However, several dentists noted keeping up with changes in guidelines as a barrier to their subsequent uptake and use. Participants expressed frustration and confusion about locating the appropriate guideline (eg, VHA, American Dental Association [ADA], and American Association of Orthopedic Surgery [AAOS]) and its correct year when making a prescribing decision. For instance, one dentist described using the various resources (Table 3, Opportunities, no. 3).

Dentists reported working with multiple nondental disciplines when prescribing antibiotic prophylaxis. In these cases, it was clear both dentists and surgeons leveraged field-specific guidelines to reduce likelihood of patient infections during outpatient dental procedures. Interestingly, dentists expressed that they were expected or asked to use national guidelines outside the ADA (Table 3, Opportunities, no. 4).

Aside from the guidance of nationally based resources, local features (ie, resources specific to the VHA) were helpful in adhering to evidence-based prescribing practices. Examples of local features came in the form of policy and decision support. For instance, having access to more complete patient information in the VHA’s integrated electronic health record system was noted as helpful in resolving, annotating, or handling conflicting requests for antimicrobial prescriptions. One dentist expressed an overall sense of relief, noting that, “the more information I have, I feel more confident in what I’m prescribing” and “if anything, it [antibiotic prescription] is more tailored” (GD-03-49).

Other dentists noted more local features more specific to their VAMC location, including access to antimicrobial stewardship expertise from pharmacies, from local policies created to help minimize conflicting opinions between medical professionals, and from the ability to readily contact medical providers within VA’s integrated health system. A variety of features were noted at the local level, all of which were mentioned influenced prescribing habits (eg, local policy to defer to physician preference). One dentist noted the benefit of involvement of an antimicrobial stewardship team (Table 3, Opportunities, no. 5).

### Social influence

Socially based influences on prescribing practices emerged, such as patient requests for antibiotics and the influence of peers and colleagues arise when making the decision to prescribe. Dentists reported several sources of social influence, some of which were helpful in making an evidence-based prescribing decision (eg, discussion with a peer), whereas other social influence(s) acted as a barrier. We differentiated between patient requests, requests from peers in dentistry, and requests from physicians (eg, primary care providers or orthopedic surgeons). Commonly, dentists reported receiving requests from nondental specialties (eg, physicians); most frequently, those outside the profession of dentistry, including orthopedic and cardiothoracic surgeons. Dentists shared that physician requests for a prescription happen infrequently; yet the social influence from nondental providers was described as a potential barrier to evidence-based prescribing, as illustrated in the following excerpt (Table 3, Opportunities, no. 6).

Most dentists reported that requests from outside medical providers and specialists for antimicrobial prescriptions were “rare” (2%–3% of the time) and “very rare” (<2% of the time) to “high” (as much as 20% of the time). However, dentists elaborated on how they respond to external requests. Interestingly, dentists from high-prescribing sites differed from dentists at low-prescribing sites in their response to external requests, fully deferring to physician requests and prescribing an antibiotic rather than ‘pushing back’ (ie, refusing to prescribe an antibiotic). The following excerpts showcase dentists’ responses to these external requests, some of which may pose a barrier to evidence-based prescribing, such as complete deferral to physician preference (Table 3, Opportunities, nos. 7–10).

Requests from patients were recorded as a rarer occurrence when compared with requests from a physician; however, dentists expressed great capability in handling these requests (Table 3, Opportunities, no. 11).

### Motivations

Motivations are the third prong of the COM-B framework theorized to influence behavior. Motivations within COM-B framework refer to both reflective and automatic processes that can influence the prescribing of antimicrobial therapies.^16,17^ These processes are thought to be internal to the individual and include reflecting on past behaviors and/or outcomes or otherwise desires, impulses, and inhibitions. Awareness of prescribing behavior and experience with severe adverse events emerged as aspects of dentists’ internal motivation in reflecting and evaluating on prior experiences as a means to make a prescribing decision and subsequent behavior.

### Awareness and severe adverse events

Overall, there was a lack of awareness in terms of the high prescribers recognizing the harm in their prescribing habits. Although several low-prescribing dentists did not explicitly mention their facility as low prescribing, there was greater awareness of both prescribing habits at their facility and ongoing quality improvement initiatives they believed improved antibiotic prescribing practices (Table 3, Motivations, no. 12).

Most dentists mentioned having little awareness of antibiotic prescribing practices outside their facility; however, dentists indicated known guideline-discordant practices within their facility(ies) and a desire to see prescribing data.

## Discussion

This study is the first to examine antibiotic prescribing using a national sample of dentists from low- and high-prescribing facilities. We identified themes regarding the processes and factors that influence dentist prescription behaviors, adding to the body of literature on outpatient dental prescribing. First, among our study findings, national guidelines were frequently mentioned as useful in prescribing; however, difficulty in locating current and relevant guidelines present a barrier to their use. Second, the social influence of physicians is a complicated area within dentistry. Although they occur infrequently, responses to physician requests harbors the potential for confusion, frustration, and conflict. Among the myriad discipline-specific guidelines, local policies can help or hinder resolution of conflict in accordance with evidence-based standards. Dentists voiced a desire for guidance that targets their practice setting, such as point-of-care guidance or local policy founded on standard of care. The social influence of physicians on dental prescribing aligns with recent research in dental prescribing^18,19^ in that prophylactic prescribing for specialty surgical patients is a unique challenge.^19,20^ Contrary to the literature, in our study, patient requests for antibiotics were cited as having little social influence on the decision to prescribe antibiotics.^21,22^ Part of the reason for this discrepancy may lie in differences due to study setting.

**Fig. 3.**
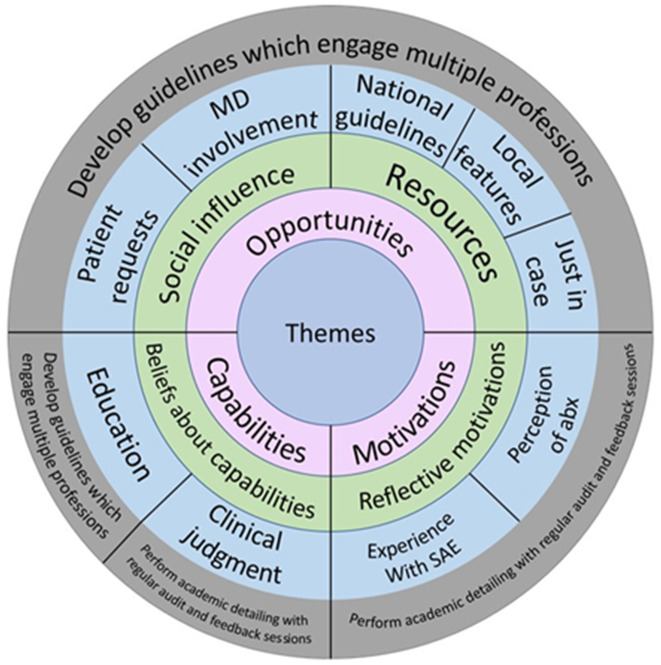
Recommended Interventions

Three key findings bear important practical implications for dental practice and antibiotic stewardship: use of guidelines, awareness of prescribing patterns, and social influence of physicians. Each key finding offers promise in selection of an appropriate dental antimicrobial stewardship strategy (Fig. 3).

First, few dentists perceived a problem with antibiotic prescribing, which held true for all dentists recruited from high-prescribing facilities. This finding reveals the need to improve awareness of prescribing patterns among high-prescribing dentists. Academic detailing offers a potential solution. Academic detailing provides periodic reporting on prescribing trends and in-depth discussion with an academic detailer; discussion can yield benefits, such as increased awareness of evidence-based prescribing guidelines, practices, and available tools to improve antimicrobial therapy selection and has been shown to improve evidence-based prescribing in nondental settings.^23^

Secondly, dentists relied upon national guidelines to inform prescribing. Yet, locating the current year, specific guideline (eg, prophylaxis for joint replacement), and issuing professional organization (eg, AHA) may prove difficult at the point of care. Antimicrobial stewardship efforts that target readily accessible decision aids for prophylaxis and treatment, such as order sets and pocket guides, should be prioritized to guide appropriate prescribing decisions for outpatient dentists.

Lastly, the social influence of physicians on antibiotic prescribing in dentistry is a multifaceted factor. Requests for antibiotics from a physician do not directly act as a barrier to evidence-based prescribing. Rather, drivers of complete deferral to physician request should be better understood. Dentists often work with nondental providers to care for the same patient(s) within a hierarchical culture.^24^ As such, a singular antimicrobial stewardship strategy may be insufficient to combat these social influences. However, preliminary steps to adherence to evidence-based antibiotic prescribing behaviors include engaging multiple professions in the development of guidelines for antimicrobial use^25^; promoting education on antimicrobial stewardship, interprofessional communication; and promoting a just culture.^22^

Fortunately, participating dentists reported both a strong belief in capability and openness to learning more about evidence-based antibiotic prescribing practices. This finding provides encouraging evidence that VA outpatient dentistry may be an area receptive to change.^11,15,26^

This study had several limitations. The 90 dentists who granted interviews provided rich contextual information. Interview approaches, however, rely on self-report of participants and offer subjective, rather than objective, insight into a phenomenon. Furthermore, this study was based on a national sample recruited at the VHA; therefore, results may not be generalizable to the dental outpatient setting in civilian practice. To illustrate, the interoperability of electronic health records as part of a large and integrated health system puts VHA dentists in the position to review medical information; thus, this setting does not fully represent private practice dentistry.

We derived 4 overarching themes from interviews with 90 dentists that speak to the factors driving antimicrobial prescribing in dentistry. Leveraging the COM-B framework, opportunities for prescribing antimicrobials appear influential in a dentist’s prescribing behavior. Future work should focus on developing and implementing antimicrobial stewardship interventions that target unique dentist prescriber needs.

## Supplementary material

For supplementary material accompanying this paper visit https://doi.org/10.1017/ash.2022.242. click here to view supplementary material
