# Supplementary material for: A qualitative approach to examining antimicrobial prescribing in the outpatient dental setting
Source: Antimicrob Steward Healthc Epidemiol. 2022 Jun 24;2(1):e102. doi: 10.1017/ash.2022.242 (PMC9726505; doi:10.1017/ash.2022.242)
Supplement: Supplementary file 1 [file ashsup.zip › S2732494X2200242Xsup001.docx]

Supplemental Appendix A

Interview Guide Example Questions [begins after audio recording has begun and demographics have been recorded outside of the interview transcript/notes]

1. How do you feel your particular VA site compares to other VAs in terms of frequency and amount of antibiotic prescribing?

*PROBE:* What makes you feel that way?

2. In your daily practice, do you use any national guidelines or VA guidance when you are making decisions around antibiotic prescribing?

*IF yes to either*, which ones do you typically use?

*IF they don’t specify a year:* Do you know the year of that guideline?

3. Over the course of your dental career, would you say that you have tended to follow changes in antibiotic guidelines or VA guidance on antibiotic prescribing or have you found that those guidelines were not that helpful in your practice?

*If response is “NOT HELPFUL”* can say more about why it wasn’t helpful.

4.What factors in your particular patient population influence the way you prescribe antibiotics?

*If they do not mention patient factors ask:* What patient factors do you considering when you prescribe antibiotics?

5. Under what circumstances, if any, do you prescribe antibiotics for patients on a “just in case” basis or “take if you have” situation? (May be referred to as “delayed prescribing”)

*Probe:* When did you begin prescribing “just in case” patients needed an antibiotic?

6. How often do you have patients asking for antibiotics when you do not think they are needed?

6b. How do you typically respond to patients who want antibiotics when you feel they are not indicated?

*Probe:* Would you be interested in learning more about how to talk to patients about antibiotics or do you think you have sufficient knowledge in that area?
