# Supplementary material for: A qualitative approach to examining antimicrobial prescribing in the outpatient dental setting
Source: Antimicrob Steward Healthc Epidemiol. 2022 Jun 24;2(1):e102. doi: 10.1017/ash.2022.242 (PMC9726505; doi:10.1017/ash.2022.242)
Supplement: Supplementary file 1 [file ashsup.zip › S2732494X2200242Xsup002.docx]

## **Appendix B.** Sampling strategy by region

| **Region** | **Facility level Rx** | **Prescriber type** | | **Prescriber level Rx** | **Prescriber type** | |
| --- | --- | --- | --- | --- | --- | --- |
|  |  | **GD** | **SP** |  | **GD** | **SP** |
| **Northeast** | High | 6 | 2 | High | 3 | 1 |
|  | Low | 2 | 3 | Low | 1 | 2 |
| **South** | High | 7 | 2 | High | 7^a^ |  |
|  | Low | 11 | 3 | Low | 5 | 1 |
| **Midwest** | High | 10 | 0 | High | 8 ^a^ | 0 |
|  | Low | 8 | 3 | Low | 2 | 0 |
| **West** | High | 18 | 5 | High | 8 | 1 |
|  | Low | 11 | 1 | Low | 9 ^b^ | 1 |

*NOTE*: GD= General Dentist, SP=Specialty Provider (e.g., oral surgeon); a= One dental prescriber from a high prescribing facility, low prescriber for region; b= one dental prescriber from a low prescribing facility, high for the region
